# Fracture of the Skull, Complicated with Rupture of the Pericardium

**Published:** 1844-12-28

**Authors:** 


### FRACTURE OF THE SKULL, COMPLICATED WITH RUPTURE OF THE PERICARDIUM.

Dr. Michon surgeon to the *Hôpital Cochin*, communicated the following case to the *Societe de Chirurgie.* A young man while at work, fell from a scaffolding thirty feet in height. Picked up, and brought to the hospital, the symptoms observed were:—coma; stertorous respiration; pulse feeble, intermittent; beatings of heart dull, and so rapid that it was impossible to reckon them; paralysis of the motility and sensibility: involuntary stools; flow of blood from the mouth and nostrils; insensibility; wound on the left side of the head; ecchymosis of the right eye; death took place two hours after.

*Sectio:* Fracture of the skull extending from the internal angle of the right orbit to the centre of the left parietal bone; sanguineous effusion in the cavity of the arachnoid; contusion of the cerebral substance near the apophysis cristæ galli. Thorax: rupture of the anterior portion of the pericardium, about seven inches in length, through which opening the heart protruded, so as to touch the lung. A deep furrow divided the part, exterior to the pericardium, from that contained in its cavity; the former was red and considerably swollen.—*Lond. Med. Times.*

